# Cardiac Findings in Amyotrophic Lateral Sclerosis: A Magnetic Resonance Imaging Study

**DOI:** 10.3389/fneur.2017.00479

**Published:** 2017-09-27

**Authors:** Angela Rosenbohm, Benjamin Schmid, Dominik Buckert, Wolfgang Rottbauer, Jan Kassubek, Albert C. Ludolph, Peter Bernhardt

**Affiliations:** ^1^Department of Neurology, University of Ulm, Ulm, Germany; ^2^Department of Internal Medicine, Kreiskrankenhaus Ehingen, Ehingen, Germany; ^3^Department of Internal Medicine II, University of Ulm, Ulm, Germany; ^4^Heart Clinic Ulm, Ulm, Germany

**Keywords:** amyotrophic lateral sclerosis, cardiac magnetic resonance tomography, cardiac involvement, sympathetic dysfunction, heart

## Abstract

The objective of this study was to investigate the potential involvement of cardiac structure and function by cardiac magnetic resonance (CMR) imaging in amyotrophic lateral sclerosis (ALS) patients. Our study included 35 patients with ALS without a history of cardiac disease and an age- and gender-matched healthy control group (*n* = 34). All subjects received a CMR in a 1.5-T whole-body scanner. Patients were also screened with Holter monitoring, echocardiography, and a blood test of cardiac markers. Myocardial mass in ALS hearts was reduced compared to the control group, and ejection volumes in the left and right heart were severely decreased in ALS patients, as shown by echocardiography and CMR. The myocardium showed increased T1 enhancement in 77% of the patients compared to 27% of controls (*p* = 0.0001). A trend toward late gadolinium enhancement patterns consistent with myocardial fibrosis was observed in 23.5% of the patients (9.1% of controls). Holter monitoring was normal in all patients as well as troponin T. Cardiac involvement seems to be present in ALS patients without clinical cardiac symptoms and with a normal cardiac routine assessment. Structural myocardial defects in CMR may be due to sympathetic dysfunction and may account for reported cardiac deaths in late-stage ALS patients.

## Introduction

A common limiting factor for life expectancy of amyotrophic lateral sclerosis (ALS) patients is respiratory failure that is caused by paresis of respiratory muscles as well as aspiration and resulting pneumonia (1, 2). Another common cause of death in ALS is sudden cardiac death (1, 3). In recent years, several studies have shown that the autonomic nervous system is also involved in ALS, since increased heart rate variability (4) and increased QTc intervals in electrocardiogram (ECG) have been reported (5). Decreased heart rate variability and a loss of correlation between blood pressure and heart rate also have been described (6, 7). Cardiac involvement has been reported in ALS by ECG, echocardiography (8), and postmortem examination (5). The involvement of the autonomic nervous system could increase the risk of heart rhythm disturbances or sudden cardiac death (9, 10). Subclinical cardiac involvement has been reported (11, 12), including non-specific ECG and echocardiographic abnormalities. In summary, cardiac involvement in ALS seems to be frequent, but systematic information on functional and structural deficits does not seem to be present.

Cardiac magnetic resonance (CMR) imaging is a valuable tool in the diagnostic assessment of inflammatory myocarditis (13–16) and for the detection of cardiac involvement in systemic muscle diseases (17, 18). The aim of this study was to characterize cardiac involvement in ALS using a comprehensive CMR protocol.

## Materials and Methods

### Patients and Controls

All patients included had possible, probable, or definite ALS according to the revised El Escorial criteria. Patients participating in the diagnostic assessment at the Department of Neurology, University of Ulm (Germany) and in the regional ALS registry (ALS Register Swabia REF) were prospectively screened and enrolled in the study. In total, 53 patients were screened and provided written informed consent. Eighteen of these patients had to be excluded due to claustrophobia or inability to lie supine because of respiratory dysfunction. The ALS Register Swabia and this CMR study were approved by the local Ethics Committee of the University of Ulm (protocols no. 11/10 and no. 05/11).

Exclusion criteria were contraindications for CMR or gadolinium-based contrast agent or pregnancy.

Blood levels of troponin T, creatine kinase (CK), myocardial creatine kinase (CK-MB), and *N*-terminal pro brain natriuretic peptide (NT-pro BNP) were analyzed in each patient. For NT-pro BNP, normative age-related values were used (19).

As controls, a cohort of age and gender matched patients was implemented. These patients exhibited no signs of cardiac insufficiency. Clinical characteristics of ALS patients and controls are presented in Table 1.

**Table 1:** Clinical characteristics of amyotrophic lateral sclerosis (ALS) patients and controls.

	ALS patients (*N* = 35)	Controls (*N* = 34)
Age (y), mean ± SD	69.54 ± 10.64	68.06 ± 9.59
Females, *n* (%)	18 (51.4)	18 (52.9)
Arterial hypertension, *n* (%)	16 (45.7)	11 (32.4)
Diabetes, *n* (%)	7 (20)	1 (3)
Body mass index mean ± SD (kg/m^2^)	25.0 ± 5.3	25.8 ± 4.2
ALS functional rating scale, points	32.4 ± 8.5	–
Forced vital capacity, %	66.11 ± 25.30	–
Spinal/bulbar, *n*	23/12	–
Creatine kinase (norm <171), U/l	244 ± 266	–
Myocardial creatine kinase (norm <25), U/l	24 ± 14	–
Troponin T (norm <14), ng/l	0 ± 0	–
N-terminal pro brain natriuretic peptide (norm 0–125), pg/ml	149 ± 206	–
Angiotensin converting enzyme inhibitor and angiotensin 1 receptor blockers, *n*	10/35	–
Beta-blocker, *n*	8/35	–
Other antihypertensive medication, *n*	4/35	–
AVB grade II, *n*	0	–
Atrial fibrillation, *n*	0	–
Supraventricular tachycardia, *n*	0	–
NSVT, *n*	0	–
VCP/h (0–100), *n*	20/26	–

*NSVT, non-sustained ventricular tachycardia; AVB, atrioventricular block; VPC/h, ventricular premature contractions/hour*.

### Holter Monitoring

A 24-h Holter monitoring was performed using a Digi Trac XT recorder (Philips Healthcare). Holter monitoring was considered abnormal in the presence of; atrioventricular block grade I–III, atrial fibrillation/flutter (AF/AFL), other supraventricular tachyarrhythmia (SVT) [>30 supraventricular premature contractions (SVPC) per hour or runs of ≥20 SVPC], frequent ventricular premature contractions (VPCs) (≥30/h), and non-sustained ventricular tachycardy (NSVT).

### Transthoracic Echocardiography

Transthoracic echocardiography was performed using a CX 50 Ultrasound (Philips Healthcare Germany). Left ventricular (LV) cavity dimensions, mass and wall thickness and diastolic dysfunction were assessed in accordance with the recommendations of the European Association of Echocardiography (20).

An abnormal echocardiography was defined by the following diameters of the left ventricle: a left ventricular end-diastolic diameter (LVEDD) >56 mm, an interventricular septum (IVS) >11 mm and a left ventricular end systolic diameter (LVESD) >40 mm. The ejection fraction was visually evaluated. In addition, echocardiography was used to assess valve disease and fractional shortening (FS).

### CMR Study

All patients were examined in a 1.5-T whole-body scanner (Intera, Philips Medical Systems, Best, The Netherlands) using a 32-channel phased-array cardiac surface coil. Steady-state free precession cine sequences were acquired in a contiguous short axis orientation covering the LV and right ventricle for volumetric and functional analysis of both ventricles (repetition time 3.4 ms, echo time 1.7 ms, slice thickness 8 mm, no interslice gap, and acquisition in end-expiration breath-hold) as previously reported (20).

Ten minutes after an intravenous application of 0.2 mmol/kg body-weight gadolinium-based contrast agent (Dotarem, Guerbet, Villepinte, France), an inversion-recovery gradient-echo sequence for evaluation of late gadolinium enhancement (LGE) was acquired in a contiguous short axis orientation covering the entire left ventricle (repetition time 7.1 ms, echo time 3.2 ms, slice thickness 8 mm, respiratory navigator, and inversion time was individually adjusted for complete nulling of the myocardium) (21).

### CMR Analysis

Cardiac magnetic resonance images (DICOM) were anonymized and transferred to a workstation. Two blinded and experienced readers (DB, PB) evaluated all images in consensus using commercially available software (cmr42, Circle, Cardiovascular Imaging, Calgary, AB, Canada). End-diastolic and end-systolic endocardial contours of the LV and right ventricle were drawn manually for evaluation of end-diastolic and end-systolic volumes, and the ejection fractions were calculated. In addition, end-diastolic LV epicardial contours were drawn for the assessment of LV myocardial mass.

For an early gadolinium enhancement compared to a thoracic skeletal muscle, early gadolinium enhancement ratio (EGEr) (14) or T1 ratio/heart/skeletal muscle (22) were chosen for analysis. In the event of gadolinium enhancement >20% in thoracic skeletal muscle, instead of analyzing a ratio an absolute value of >45% was used as cut-off for myocardial enhancement (23). For a T1 ratio, values >4 were considered to be increased. These criteria are well established and suggested for CMR diagnostic assessment of inflammatory myocardial disease (14). Increased myocardial T1 enhancement consists of either a pathological T1 ratio or an EGEr >45%.

The inversion-recovery gradient-echo sequence was evaluated for the presence of a hyper-enhancement consistent with myocardial fibrosis. LGE volume was quantified as a percentage of the LV myocardial mass using a cutoff signal intensity increase of more than five SDs of remote myocardium (21). An LGE pattern was globally assessed as ischemic with a subendocardial and/or transmural enhancement and non-ischemic with subepicardial and/or intramural enhancement.

### Statistical Analysis

All data are reported as a mean value ± SD in comparison to the control group. An unpaired *t*-test was used for the cohort comparison. A *p*-value ≤0.05 was regarded as statistically significant.

## Results

Twelve patients had bulbar onset and spinal onset was seen in 23 cases. Mean disease duration at CMR was 2.6 ± 2.2 years and mean ALS functional rating scale (ALSFRS)-R score was 32.4 ± 8.5 points. None of the patients had any cardiac complaints. Patients were characterized with respect to cardiovascular risk factors, antihypertensive co-medication, and vital capacity as indicated in Table 1. Holter monitoring was performed on 26 patients and did not show any abnormalities. Healthy controls were clinically characterized for arterial hypertension (AHT) and diabetes.

### Holter Monitoring

In particular, we did not find any abnormality on Holter monitoring in patients with ALS (*n* = 26), with regard to atrial fibrillation/flutter, supraventricular tachycardia, atrioventricular block, and non-sustained ventricular tachycardia.

Several premature ventricular contractions were monitored (Table 1). Overall, no association was observed between abnormal myocardial CMR (T1 Ratio, LGE) and abnormal findings on Holter monitoring.

### Echocardiography

Echocardiography revealed normal diameters of the ventricles. The systolic function was not diminished in the visual evaluation with normal values of FS. Slight insufficiencies of the aortic, tricuspidal and mitral valve were reported in several patients. All other documented values were in the normal range (Table 2). No pericardial effusions were reported.

**Table 2:** Results of echocardiography in 30 amyotrophic lateral sclerosis (ALS) patients.

	ALS patients
LA (norm <40), mean ± SD (mm)	37.7 ± 6.4
LVEDD (norm <56 mm), mean ± SD (mm)	46.2 ± 4.6
LVESD (norm <42 mm), mean ± SD (mm)	26.2 ± 4.0
FS fractional shortening (LVEDD–LVESD) (norm > 25%), mean ± SD (%)	40.4 ± 6.6
IVSDD (norm 5–11 mm), mean ± SD (mm)	9.6 ± 2.0
AI (slight), *n*	7/30
MI (slight), *n*	13/30
TI (slight), *n*	15/30

*LA, left atrium; LVEDD, left ventricular end-diastolic diameter; LVESD, left ventricular end systolic diameter; FS, fractional shortening; IVSDD, intraventricular septum end-diastolic diameter; AI, aortic insufficiency; MI, mitral insufficiency; TI, tricuspidal insufficiency*.

### Blood Tests

Creatine kinase was elevated in 14/35 patients (mean 239 ± 265 U/l, normal range <171 U/l) and CK-MB in 6/22 (mean 24 ± 14 U/l, normal range <25 U/l). Troponin T was normal in all patients tested (*N* = 28). NT-pro BNP (with age- and gender-correlated normative values) was abnormal in 3/35 patients, indicating myocardial insufficiency. One of these patients showed a previous myocardial ischemia and the other two showed pericardial effusions.

### CMR Data

Cardiac magnetic resonance was completed in 35 ALS patients and 34 controls. Mean left and right ventricular ejection fractions (LVEF and RVEF) were in the normal range (Table 3), but myocardial mass and myocardial volumes were significantly reduced in ALS patients compared to the control group (LV stroke volume, left ventricular end-diastolic volume (LVEDV), ventricular mass, right ventricular stroke volume, and right ventricular end-diastolic volume). *N* = 27 ALS hearts had LVEF >60% of which *n* = 18 had increased T1 enhancement (67%) compared to *N* = 22 normal control hearts of which 6 had increased T1 enhancement (27%).

**Table 3:** Cardiac magnetic resonance (CMR) characteristics of 35 ALS cases.

	Unit	ALS cohort *n* = 35	Control cohort *n* = 34	Unpaired *t*-test *p*-value
Age (years)	Mean ± SD	69.54 ± 10.64	68.06 ± 9.59	0.55
Females, *n* (%)	Frequency	18 (51.4)	18 (52.9)	0.90
AHT, *n* (%)	Frequency	16 (45.7)	11 (32.4)	0.26
Diabetes, *n* (%)	Frequency	7 (20)	1 (3)	**0.03**
LVEDV (ml)	Median (5; 95)	99 (68; 158.2)	136 (82.4; 213.6)	**0.0001**
LVEDV index (ml/m^2^)	Median (5; 95)	57.74 (29.09; 88.14)	73.18 (42.78; 110.6)	**<0.0001**
LVSV (ml)	Mean ± SD	68.30 ± 17.62	82.09 ± 20.66	**0.0046**
LVSV index (ml/m^2^)	Mean ± SD	37.05 ± 9.737	44.75 ± 10.34	**0.0024**
Ventricular mass (g)	Mean ± SD	79.38 ± 26.16	99.0 ± 27.30	**0.0047**
Ventricular mass index (g/m^2^)	Mean ± SD	41.98 ± 16.04	50.3 ± 17.36	**0.0435**
LVEF (%)	Median (5; 95)	64 (55.2; 74.7)	62 (44.4; 75.6)	**0.034**
RVEDV (ml)	Median (5; 95)	96 (67; 163.65)	134 (68.6; 219.2)	**0.0005**
RVEDV index (ml/m^2^)	Median (5; 95)	55.63 (27.35; 88.23)	77.63 (35.82; 115.7)	**<0.0001**
RVSV (ml)	Mean ± SD	65.16 ± 18.59	82.59 ± 21.87	**0.001**
RVSV index (ml/m^2^)	Mean ± SD	35.53 ± 10.24	45.00 ± 11.06	**0.0006**
RVEF (%)	Median (5; 95)	64 (55.15; 78.4)	60 (45.4; 80.8)	0.081
T1-ratio	Median (5; 95)	4.8 (2.0; 14.13)	3.6 (1.8; 10.6)	0.076
T1 path., *n* (%)	Frequency	24/31 (77.4)	8/30 (26.7)	**0.0001**
PE, *n* (%)	Frequency	7 (21.9)	6 (17.6)	0.67
LGE, *n* (%)	Frequency	8 (23.5)	3 (9.1)	0.11

*AHT, arterial hypertension; LVEDV, left ventricular end-diastolic volume; LVSV, left ventricular stroke volume; LVEF, left ventricular ejection fraction; RVEDV, right-ventricular end-diastolic volume; RVSV, right-ventricular stroke volume; PE, pericardial effusion; LGE, late gadolinium enhancement*.

*Significant *p* values < 0.05 are marked in bold. CMR indexes related to body surface areas (BSA), which was calculated by the Dubois and Dubois regression formula*:

*BSA = 0.007184 × weight (kg)^0.425^ × height (cm)^0.725^*.

Reduced LV function (ejection fraction <60%) was observed in 6/33, and five of these patients also showed increased T1 enhancement. *N* = 12 controls had reduced LVEF, 2 of them had increased T1 ratio.

**Figure 1:**
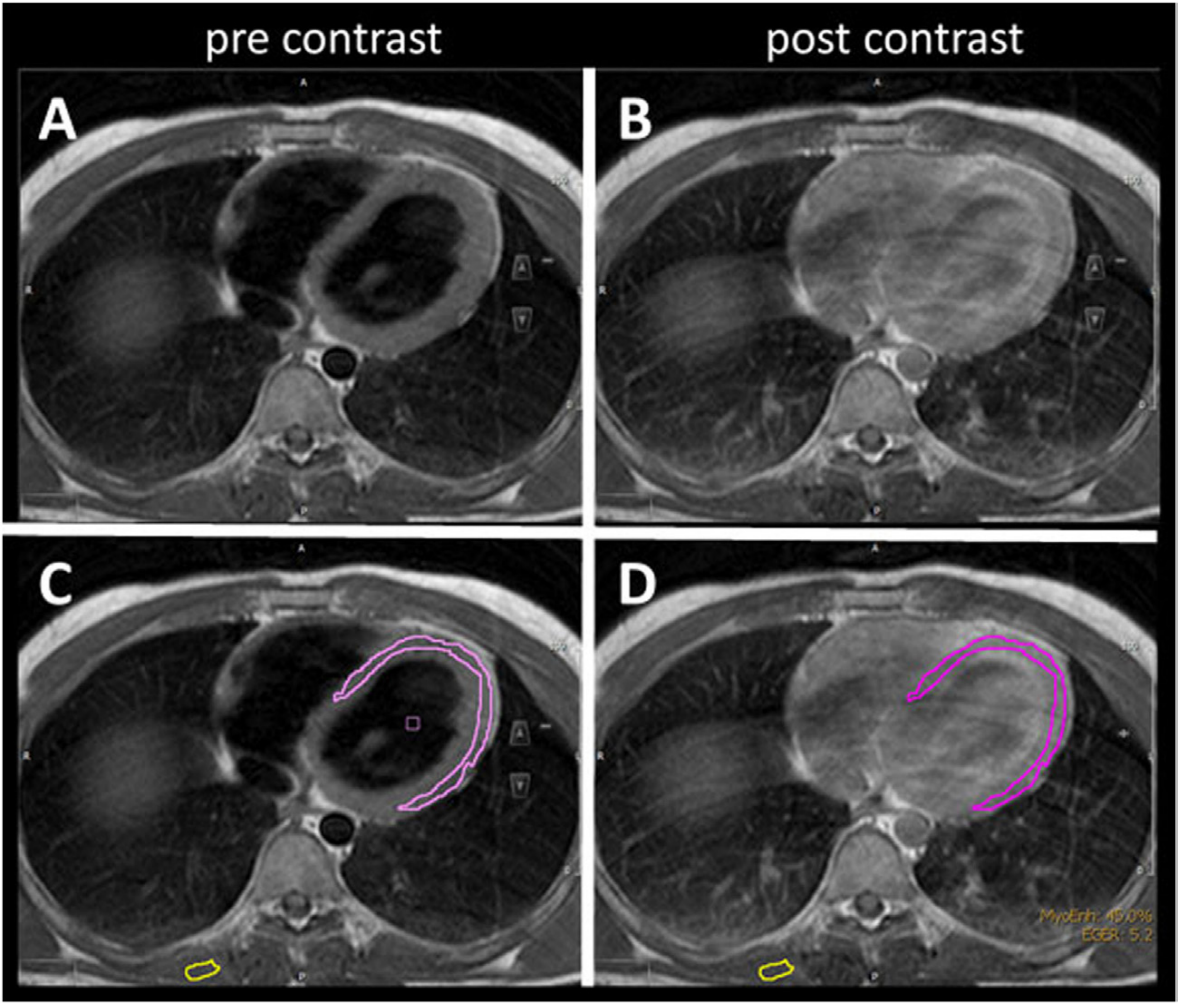
T1 turbospinecho sequences in the transversal position before **(A,C)** and after **(B,D)** gadolinium administration. For quantification of early gadolinium enhancement, contours of the left ventricular myocardium (pink) and skeletal muscle (yellow) were manually delineated **(C,D)** in the pre- and post-contrast images.

The mean LVEF in ALS patients was 64% (range 52–80) compared to controls (mean 60%, range 27–85). Early myocardial gadolinium enhancement in T1 could be observed in 24/31 (77%) of ALS patients (Figure 1) and in 8/30 controls, which was statistically different (*p* = 0.0001).

**Figure 2:**
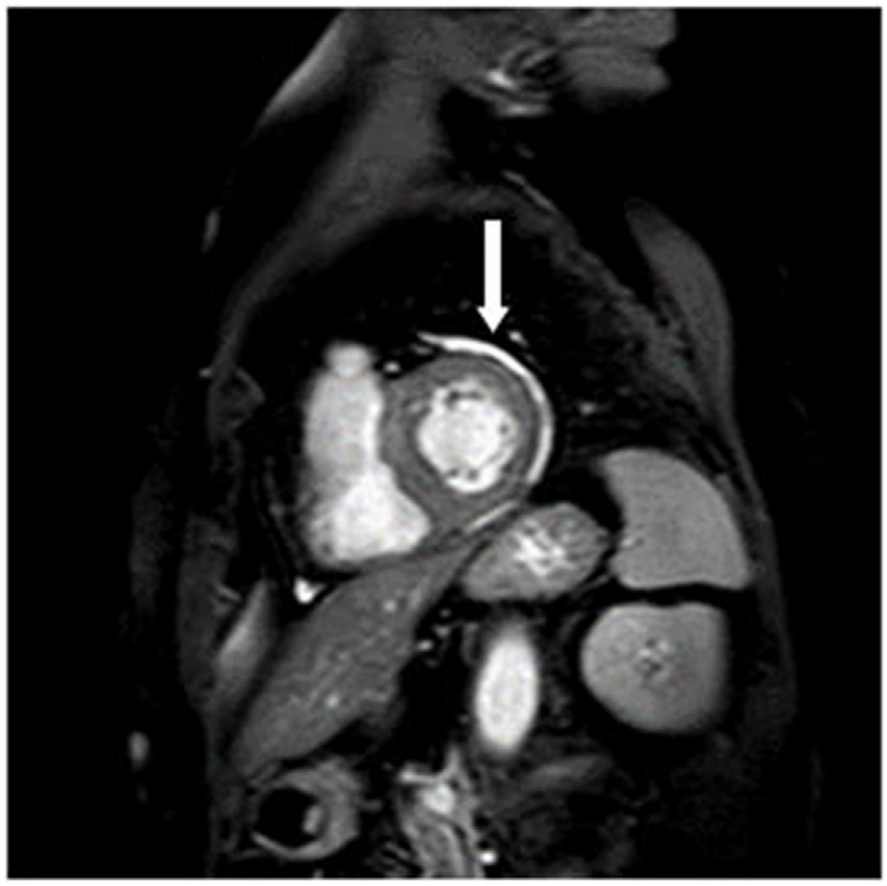
Pericardial effusion (arrow) in short axis view.

8/35 patients had a pericardial effusion without a hemodynamic restriction (Figure 2) which was also detected in 6/34 controls. There was no significant difference from healthy controls. The detected pericardial effusions in the CMR were not visible in the echocardiography.

**Figure 3:**
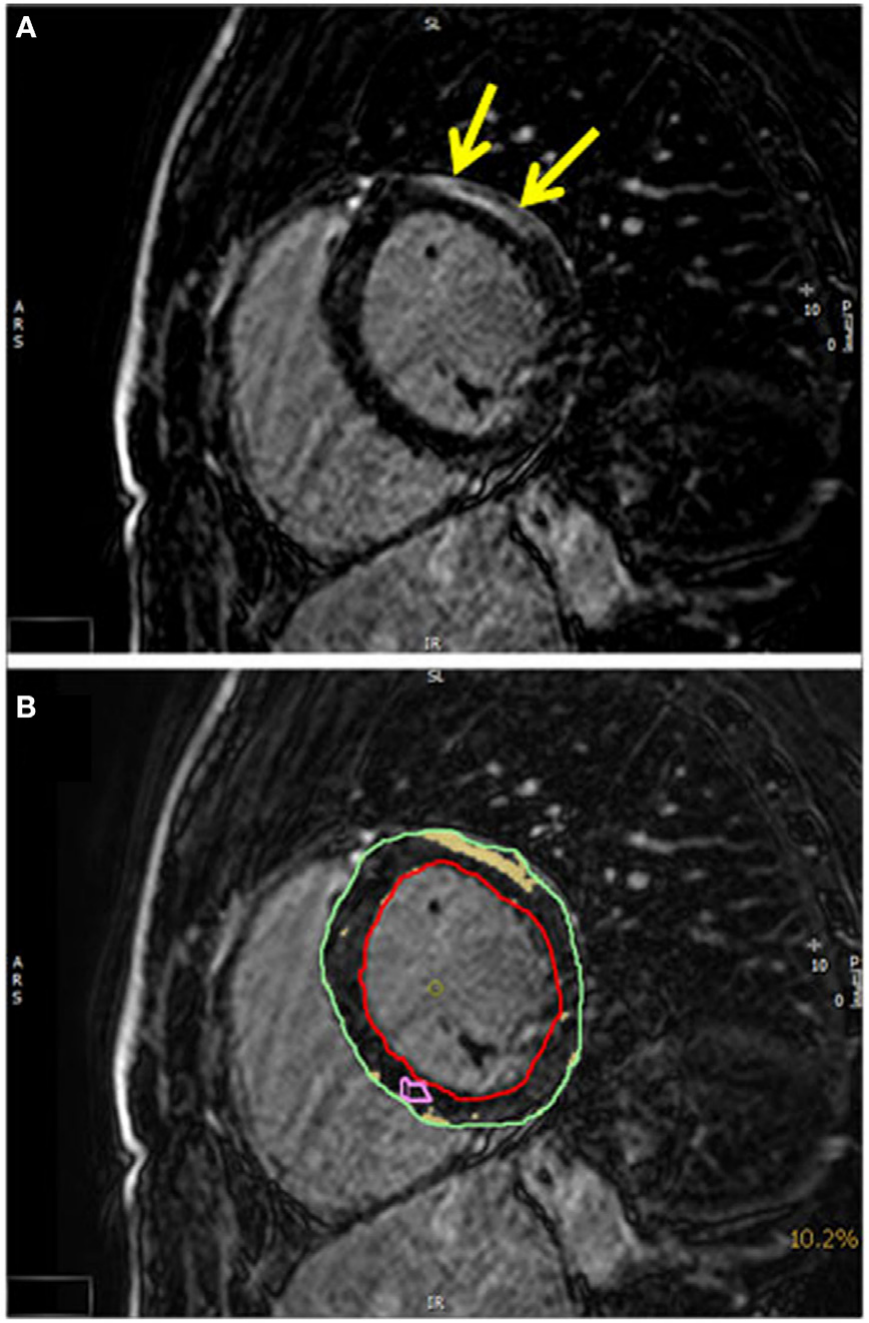
**(A)** A T1-weighted inversion-recovery sequence in short axis view showing epicardial late enhancement in the anterolateral myocardial segments (yellow arrows). **(B)** The quantification of the late enhancement area (yellow) in relation to the myocardial mass (epicardial contour green and endocardial contour red).

Late gadolinium enhancement patterns consistent with a myocardial fibrosis were detected in eight (23%) patients, but this abnormality did not reach significance, since three controls also had a pathological LGE. In four ALS patients, LGE was distributed in the basal inferolateral segments (Figure 3). In one patient, LGE was observed to be localized inferoseptally. These LGE patterns are not characteristic for an ischemic origin, but are commonly reported in inflammatory myocarditis or cardiac involvement in systemic diseases (13, 18).

A comparison of ALS patients with and without LGE and with or without increased T1 ratio did not show any statistical difference concerning heart function parameters and clinical severity (Table 4).

**Table 4:** A comparison of amyotrophic lateral sclerosis patients with and without late gadolinium enhancement (LGE) or increased T1 enhancement concerning heart function parameters and clinical severity.

	With LGE	Without LGE	Unpaired *t*-test LGE	With increased T1 ratio	Without increased T1 ratio	Unpaired *t*-test T1 ratio
Cases, *N* (%)	8 (23.5)	26 (76.4)	**0.0063**	24 (77.4)	7 (22.6)	**<0.0001**
Mass, g (mean ± SD)	77 ± 31	81 ± 25	0.5366	77 ± 33	76 ± 18	0.9446
LVEF% (mean ± SD)	63.9 ± 5.4	65.0 ± 6.2	0.5725	61.8 ± 8.3	64.1 ± 5.6	0.4910
RVEF% (mean ± SD)	67.9 ± 11.6	63.5 ± 6,2	0.0676	59.3 ± 10.7	62.3 ± 6.4	0.4913
T1-ratio (Norm <4) (mean ± SD)	5.4 ± 3.7	5.8 ± 3.7	0.8907	–	–	–
LGE, *n* (%)	–	–	–	3 (13)	1 (14)	0.9053
ALSFRS-R (points) (mean ± SD)	33.5 ± 11.1	32.0 ± 7.8	0.6676	31.6 ± 8.6	32.4 ± 8.7	0.8235
ALS duration (years) (mean ± SD)	1.4 ± 0.7	3.0 ± 2.3	0.0775	2.4 ± 1.9	3.6 ± 2.9	0.2136

*Significant p values < 0.05 are marked in bold*.

## Discussion

As the rather frequent occurrence [10% according to Ref. (3)] of cardiac deaths in ALS is not well understood (1), this study was performed in order to characterize myocardial tissue by CMR in ALS patients without any cardiac complaints. No significant abnormalities in the routine cardiac assessment with Holter monitoring and echocardiography could be found except slight valve insufficiencies in up to half of the patients. Recent studies have shown that CMR is an important diagnostic tool for detecting cardiac involvement in systemic diseases, such as cardiomyopathies and amyloidosis (22, 24–26). Abnormalities in CMR were frequent in ALS, in particular early gadolinium enhancement (77%) as a parameter of enhanced extracellular volume, that is either capillary leakage or fibrosis. Pericardial effusions were detected in 22% and LGE in 23.5% as a marker of diffuse myocardial fibrosis. The latter abnormalities did not differ significantly from controls (17.6% effusions and 9.1% LGE). None of the patients displayed any characteristics of cardiac ischemia that could account for the observed myocardial changes (ischemic pattern with a subendocardial and/or transmural enhancement).

In comparison with healthy controls, ALS patients showed significantly lower left and right ventricular volumes and ventricular mass. Since the heart scales with the size of the body and, therefore, with height and weight, we used the index of ventricular mass and volumes as a ratio to body surface area (BSA) for comparison with controls (Index = mass/BSA or volume/BSA) (27).

Even after indexing these values to BSA, which may be lower in ALS patients, these differences remained. ALS hearts had lower myocardial mass than control hearts, and ejection volumes in the left and right heart were decreased in ALS patients. It is possible that reduced body weight or reduced physical activity may account for these abnormalities. There are studies implying that BMI is a strong predictor for heart mass (28), but our control group did not differ with regard to BMI. Reduced muscle mass or malnutrition, therefore, are not the cause for loss of myocardial mass in our cohort, as also shown by the statistically different indexes in Table 3.

The function and the size of the heart show a regression in healthy inactive persons even without any structural heart disease (29). Therefore, we assume that significantly reduced heart mass and ejection volumes are a consequence of more sedentary/inactive behavior in ALS patients if compared to healthy adults.

At a higher age, even healthy persons show alterations in CMR as can be observed in our age-matched control cohort. Increased myocardial T1 values or LGE may be attributed to unrecognized cardiac events (myocarditis, infarction) and hypertension (30, 31). Since the frequency of diabetes differed significantly in cases and controls (*p* = 0.03), some of the reported abnormalities in ALS patients might be due to diabetic complications. With respect to CMR, a subclinical diastolic dysfunction but no significant fibrosis in LGE has been reported in diabetes (32). With respect to ALS, this finding has to be seen in the context of the association between diabetes (type 2) and ALS as previously reported (33, 34), but the data of our study cannot add substantially to this discussion.

With respect to the origin of the detected increased myocardial T1 values, typical patterns of myocardial infarction [subendocardial or transmural LGE pattern (35)] were not detected. In a disease with denervation as the main muscular pathology, early gadolinium enhancement is likely to be associated with denervation edema (36–38).

One explanation for denervation would be increased sympathetic activity induced by respiratory weakness and, therefore, pulmonary hypertension, but no signs of pulmonary hypertension could be detected in our patient cohort. Primary involvement of the autonomic cardiac nerves may be an explanation for postulated sympathetic hyperactivity. Several groups have detected evidence of sympathetic hyperactivity even at the time of the initial ALS diagnosis (9, 39, 40). Additional studies reported primary sympathetic-impaired autonomic control in ALS patients by ECG abnormalities (6, 41). In a SOD1 mouse model of ALS, preganglionic sympathetic denervation was histologically confirmed (42). Tamsulosin hydrochloride as a selective alpha 1-adrenoceptor blocker was shown to be useful for suppressing central sympathetic hyperactivity but it was not tested for the long-term outcome of ALS patients (43).

Studies of pTDP-43 pathology in the brain and the associated neuronal loss have revealed involvement of amygdala and the hypothalamus as central sympathetic structures (44, 45).

Given that sympathetic hyperactivity can be associated with stress-induced cardiomyopathy and sudden cardiac death, this hypothesis may explain why sudden cardiac death is one of the main causes of death in ALS after respiratory insufficiency.

Laboratory parameters have shown increased level of CK, as expected in ALS patients, whereas troponin T as an acute cardiac marker was not increased. Heart failure leading to increased NT-pro BNP was rarely detected and could be attributed to (preexisting) structural defects. Therefore, CMR seems to be more sensitive for the detection of cardiac involvement in ALS than cardiac parameters in blood tests.

Limitations of this study are the inability to differentiate the gadolinium enhancement with regard to the underlying pathophysiology. Furthermore, our results probably underestimate cardiac changes due to relatively unaffected respiration of the ALS participants, since severely affected ALS patients could no longer lie flat in CMR.

In summary, functional cardiac involvement with a tendency toward lower ejection volumes in ALS hearts and an increase in early myocardial T1 contrast enhancement appears to be a common finding in ALS. The late enhancement distribution did not correspond to typical ischemic patterns with subendocardial involvement. Hence, the changes are unlikely due to cardiac ischemia and do not correlate with the clinical severity of ALS. We suggest that the most likely mechanism is a primary dysfunction of sympathetic heart regulation. If sympathetic hyperactivity is the cause for these alterations, therapeutic beta- or alpha-blocking strategies should be considered and might be studied in future clinical trials.

## Ethics Statement

This study was carried out in accordance with the recommendations of the Ethics Committee of the University of Ulm with written informed consent from all subjects. All subjects gave written informed consent in accordance with the Declaration of Helsinki. The protocol was approved by the Ethics Committee of the University of Ulm (protocols no. 11/10 and no. 05/11).

## Author Contributions

AR: acquisition of data, data analysis, writing of the manuscript draft, study supervision, and critical revision of manuscript. BS: acquisition of data, data analysis, interpretation of data, and writing of the manuscript draft. DB: acquisition of data, data analysis, writing of the manuscript draft, and critical revision of manuscript. JK: interpretation of data and critical revision of manuscript. WR: study concept and design, study supervision, and critical revision of manuscript. PB and AL: study concept and design, interpretation of data, study supervision, and critical revision of manuscript.

## Conflict of Interest Statement

The authors declare that the research was conducted in the absence of any commercial or financial relationships that could be construed as a potential conflict of interest.
